# Therapeutic Potential of Leptin in Neurodegenerative Disease

**DOI:** 10.3390/biomedicines13122969

**Published:** 2025-12-03

**Authors:** Jenni Harvey

**Affiliations:** Division of Neuroscience, School of Medicine, University of Dundee, Dundee DD1 9SY, UK; j.z.harvey@dundee.ac.uk; Tel.: +44-1382-383359

**Keywords:** leptin, hippocampus, amyloid, synaptic plasticity, tau

## Abstract

Alzheimer’s disease (AD) is an age-related neurodegenerative disorder, characterised by the build-up of amyloid beta (Aβ) plaques and neurofibrillary tangles comprising hyper-phosphorylated tau. Increasing evidence indicates that in the early stages of AD, elevated levels of oligomeric forms of Aβ and phosphorylated tau (p-tau) gives rise to impaired synaptic function which ultimately drives AD-associated cognitive abnormalities. Thus, developing drugs that can limit the synaptic impairments that occur early in AD may have therapeutic benefits. Clinical evidence increasingly supports a link between lifestyle choices and AD risk. Indeed, there is an association between the circulating levels of the metabolic hormone leptin, mid-life obesity and disease risk, which has in turn stimulated interest in targeting the leptin system to treat AD. It is well-established that leptin readily accesses the brain, with the hippocampus, a key region that degenerates in AD, identified as a prime target for this hormone. Within the hippocampus, leptin has cognitive enhancing properties as it markedly influences the cellular events underlying hippocampal-dependent learning and memory, with significant impact on synaptic plasticity and trafficking of glutamate receptors at hippocampal excitatory CA1 synapses. Moreover, studies using a range of cell-based systems and animal models of disease indicate not only that leptin has powerful pro-cognitive effects, but also that leptin protects against the unwanted synapto-toxic effects of Aβ and tau, as well as enhancing neuronal cell viability. Moreover, recent studies have demonstrated that smaller leptin-based molecules replicate the full repertoire of protective features of whole leptin. Here we review the evidence that the leptin system is a potential novel avenue for drug discovery in AD.

## 1. Introduction

Leptin, the obese (ob) gene product, is an important metabolic hormone that is primarily made in white adipocytes. The circulating levels of leptin are directly proportional to body fat stores [1], however, daily fluctuations in leptin levels arise in response to altered feeding status. Peripherally derived leptin is readily transported across the blood–brain barrier into the brain where it targets the key hypothalamic nuclei involved in regulating energy homeostasis. Leptin receptors (LepRs), which comprise six distinct LepR isoforms (LepRa-f), mediate the effects of leptin. LepRb, which is known as the long form due to its extended C-terminal, is the main isoform with signalling capacity. Like other class I cytokines [2], leptin binding to LepRs drives the homo-dimerization of the receptor, leading to phosphorylation and activation of janus tyrosine kinase 2 (JAK2), which in turn stimulates different signalling pathways. In neurons, the activation of LepRs drives JAK2-signal transducers and activators of transcription (STAT3) as well as phosphoinositide 3-kinase (PI 3-kinase) and ERK signalling [3].

It is well-documented that LepRs are distributed throughout the brain. In line with leptin’s role in regulating food intake and body weight, LepRs are highly concentrated in hypothalamic regions that control energy homeostasis [4,5]. However, high levels of LepR expression have been detected in limbic regions, particularly within the hippocampus [4,6]; a brain region that is critically involved in learning and memory processes [7]. Using dual-labelling immunocytochemical techniques, Shanley et al. [8] were the first to probe the subcellular distribution of LepRs and verified LepR expression at hippocampal synapses. Subsequent studies also identified LepRs expression at cerebellar synapses [9]. There is good evidence for the expression of LepRs at excitatory synapses as LepR co-localises with GluN2A-containing NMDA receptors (NMDARs) [10], which points towards leptin playing a potential regulatory role at hippocampal excitatory synapses. Indeed, widespread evidence supports this notion, as leptin has been shown to markedly influence synaptic transmission at hippocampal CA1 synapses [3].

## 2. Regulation of Hippocampal Excitatory Synaptic Function by Leptin

Activity-dependent changes in the strength of hippocampal excitatory synapses are known as synaptic plasticity. Two of the most well-documented forms of synaptic plasticity are long-term potentiation (LTP) and long-term depression (LTD); processes that are critically involved in hippocampal-dependent learning and memory [7]. Numerous reports have identified that alterations in the functioning of leptin significantly impact hippocampal synaptic plasticity. Indeed, impairments in both LTP and LTD have been observed in obese rodents (*db*/*db* mice; *fa*/*fa* rats) that are leptin-insensitive due to lepR mutations [11,12]. In line with the role of synaptic plasticity in memory function, *db*/*db* mice and *fa*/*fa* rats also exhibit deficits in their ability to perform hippocampal-dependent memory tasks [13,14]. Thus, studies in *db*/*db* mice and *fa*/*fa* rats have identified impaired performance in the Morris water maze, indicating deficits in hippocampal-dependent spatial memory. Moreover, obese leptin-insensitive (*fa/fa*) rats exhibit diminished ability to undertake the variable-interval delayed alternation task, suggesting deficits in hippocampal-dependent memory. Direct hippocampal administration of leptin in wild type rodents is also reported to result in the facilitation of synaptic plasticity, and it improves performance in hippocampal-dependent memory tasks [15,16]. Thus, a bi-lateral administration of leptin into the hippocampus facilitates performance in the T-maze footshock avoidance task [15], whereas an intravenous application of leptin into wild-type rodents leads to improved performance in passive avoidance and spatial memory tasks [17]. Collectively, these findings indicate that leptin boosts hippocampal learning and memory and therefore leptin acts as a potential cognitive enhancer.

Within the hippocampus, two distinct excitatory inputs converge on CA1 pyramidal neurons. The most well-studied is the Schaffer-collateral (SC) input which forms part of the classical tri-synaptic hippocampal circuitry. The other input to CA1 neurons is the temporoammonic (TA) input, which involves direct innervation by the entorhinal cortex. Activity-dependent synaptic plasticity at TA-CA1 synapses is implicated in episodic memory, as well as spatial memory [18,19]. Evidence is growing that leptin can modify the efficacy of excitatory synaptic transmission at both SC-CA1 and TA-CA1 synapses [20,21,22].

Electrophysiological studies by Shanley et al. [23] first showed that the direct application of leptin to hippocampal slices drives the conversion of short-term potentiation (STP) into LTP. Subsequent studies established that acute exposure to leptin resulted in the induction of a novel form of LTD, and it can reverse (or de-potentiate) LTP at juvenile SC-CA1 synapses [24,25]. Conversely, at adult SC-CA1 synapses, leptin induces a sustained increase in synaptic efficacy (leptin-induced LTP) that persists after leptin washout [22]. A common feature in these studies is that NMDA receptor activation is pivotal for leptin’s effects on synaptic efficacy at SC-CA1 synapses. Thus, the ability of leptin to regulate synaptic transmission is completely inhibited following the blockade of NMDA receptors with D-AP5 [20,22,26]. Interestingly, the polarity of leptin’s effects on hippocampal synaptic efficacy is highly dependent on the subunit composition of NMDA receptors. For instance, the leptin-driven depression of synaptic transmission at SC-CA1 synapses early in postnatal development involves the activation of GluN2B-containing NMDA receptors as the effects are blocked by ifenprodil [26]. In contrast, in an adult hippocampus, the persistent increase in synaptic transmission induced by leptin depends on GluN2A subunits as prior treatment with NVP-AAM077, an NMDA receptor antagonist with preferential selectivity for GluN2A subunits [26,27]. The activation of NMDA receptors with distinct molecular identity also mediates the effects of leptin at TA-CA1 synapses. Thus, in a juvenile hippocampus (P14–21), the ability of leptin to induce LTP at TA-CA1 synapses requires stimulation of GluN2B-containing NMDA receptors, whereas at adult TA-CA1 synapses GluN2A-containing NMDA receptors are pivotal for the persistent synaptic depression induced by leptin [21,28].

There is now good evidence that NMDA-dependent synaptic plasticity at TA-CA1 synapses is pivotally involved in learning and memory [18,19]. Moreover, increasing evidence indicates that TA-CA1 synapses are regulated by various neuromodulators, which in turn influences not only synaptic function and memory, but also other higher brain functions. Indeed, TA-CA1 synapses are regulated by corticosterone and serotonin, and this plays a pivotal role in mediating the synaptic alterations associated with chronic stress [29,30]. In line with TA-CA1 synapses being a target for modulation, leptin regulates the functioning of TA-CA1 synapses at different stages of development. In juvenile tissue, the application of leptin results in a novel form of NMDA-dependent TA-CA1 LTP [20] whereas in adult tissue, leptin gives rise to a persistent synaptic depression [21,28]. Furthermore, divergent cellular mechanisms underlie the opposing effects of leptin at TA-CA1 and SC-CA1 synapses, as leptin-induced TA-CA1 LTP requires PI3-kinase, whereas ERK signalling is implicated in synaptic depression induced by leptin at SC-CA1 synapses [3]. Interestingly, in occlusion studies, activity-dependent synaptic plasticity at TA-CA1 synapses has been found to display similar expression mechanisms to the persistent synaptic changes induced by leptin [20,21].

## 3. Leptin Regulates the Trafficking of Glutamate Receptors

In line with the pivotal role of NMDA receptors in mediating leptin’s effects, there is now significant evidence from cellular studies in primary neurons and brain slices, that exposure to leptin facilitates NMDA-induced responses in the hippocampus [23,26]. Moreover, in two electrode voltage clamp studies performed in *Xenopus* oocytes expressing a combination of LepRs and GluN1/GluN2A NMDA receptors, NMDA-induced currents are significantly larger after treatment with leptin [23,31]. Interestingly, in *Xenopus* oocyte studies, leptin increased the magnitude of the inward currents induced by maximal concentrations of NMDA. This suggests that leptin rapidly alters the density of plasma membrane NMDA receptors and therefore leptin may regulate the trafficking of NMDA receptors [31]. In agreement with this, recent studies by Bland et al. [32] have revealed that the formation of glutamatergic synapses is regulated by leptin, and that the capacity of leptin to boost trafficking of NMDA receptor contributes to synaptogenesis.

In addition to controlling the NMDA receptor trafficking, the mobility of AMPA receptors to and away from synapses is also modified by leptin. Thus, in adult hippocampal slices, the ability of leptin to induce SC-CA1 LTP is coupled to an increase in the rectification of synaptic AMPA receptors. This suggests that the leptin-driven persistent increase in synaptic strength is due to the synaptic insertion of GluA2-lacking AMPA receptors [22]. Prior treatment with philanthotoxin, an inhibitor of GluA2-lacking AMPA receptors, prevented the effects of leptin, thereby supporting the notion that leptin drives the delivery of these AMPA receptors to synapses. These findings are consistent with the pivotal role that AMPA receptor trafficking plays in hippocampal synaptic plasticity [33], but there are also parallels with the transient alterations in the molecular composition of synaptic AMPA receptors observed after the induction of NMDA-dependent LTP [34]. Studies in primary hippocampal neurons [22] support these electrophysiological findings, as the exposure to leptin augments the plasma membrane and synaptic expression of the AMPA receptor subunit, GluA1. Moult et al. [22] further explored the cellular mechanisms involved downstream of LepRs and identified a key role for the inhibition of the phosphatase PTEN. As PTEN promotes dephosphorylation of PIP_3_ to PIP_2_, the inhibition of the PTEN function would ultimately lead to an increase in intracellular PIP_3_ levels. Consequently, it is likely that leptin-driven inhibition of PTEN leads to an elevation in intracellular PIP_3_ levels, which in turns promotes the insertion of GluA2-lacking AMPA receptors into synapses [22]. Collectively, the ability of leptin to modify glutamate receptor trafficking, combined with its persistent effects on excitatory synaptic efficacy at CA1 synapses, suggests that it has potential cognitive enhancing actions.

## 4. Leptin and Neurodegenerative Disease

Evidence from clinical studies indicates that key features of human cases of Alzheimer’s disease (AD) and related dementias are deficits in cognition and memory. Although most AD cases are sporadic, increasing evidence indicates that lifestyle choices contribute significantly to AD risk later in life [35,36]. Recent studies indicate an association between circulating leptin levels and AD. Moreover, clinical findings indicate that obesity in mid-life markedly influences AD risk [37,38]. The association of body weight with the development of AD indicates the possible involvement of leptin and other adipokines, as adipose tissue is the prominent site for generation of various hormones involved in energy homeostasis control [39]. Consequently, as an obese phenotype is caused by leptin resistance, it is likely that alterations in the functioning of leptin and/or leptin resistance later in life contribute to the development of AD. Indeed, attenuated plasms leptin levels are common in AD patients [40], and clinical evidence has identified correlations between weight loss and the progression of AD [41]. A prospective study of the Framingham cohort observed much lower incidence of AD in non-obese individuals with high leptin levels, than those with reduced leptin levels [42]. Recent studies have also identified a correlation between plasma leptin levels and Aβ levels, suggesting molecular interplay between the leptin metabolism and brain amyloid deposition [43]. Furthermore, functional imaging studies of brain atrophy in healthy middle-aged adults observed that the higher bioavailability of leptin was associated with greater protection of brain white matter, indicating that in mid-life, elevated blood leptin bioavailability may protect against the risk of dementia [44]. However, not all studies have observed an association between leptin and impaired cognitive function or have found any change in plasma leptin levels in AD patients [45,46], indicating that leptin’s role is liable to be complex. It is likely that the overall AD risk involves the consideration of a combination of potential causative factors, including interactions between leptin and other metabolic hormones, as well as any underlying metabolic disease.

Although there are complexities around the causative role of leptin in human cases of AD, several studies have explored the role of leptin in various animal models of AD. Thus, in APPSwe and CRND8 mice, the circulating levels of leptin are markedly less than their wild type littermates at the same age [15,47], suggesting an impaired metabolic function in AD. Moreover, treatment with leptin reversed the AD-related cognitive impairments in the AD mouse models [15], indicating not only that these deficits are leptin-dependent but also that it is feasible that dysfunctions in the leptin system contribute to the development of AD in rodents. Recent studies have detected altered leptin and LepR brain expression in the 5XFAD rodent model of AD [48], which also supports the motion that impaired leptin and/or lepR-driven signalling may be involved in AD in rodents.

## 5. Leptin Has Protective Actions in the CNS

Since the early studies that uncovered that leptin deficiencies resulted in reduced brain weight due to decreased neuronal viability [49], it is now well-established that leptin has protective actions in the CNS. Indeed, numerous reports have confirmed that leptin can promote neuronal survival as well as limiting the degree of neuronal cell death [50,51]. Recent evidence has implicated dynamic alterations in mitochondrial structure and function in mediating leptin’s protective actions. Thus, leptin protects the hippocampal neurons against glutamate-induced excitotoxicity by stabilising the mitochondrial membrane [52]. Furthermore, the treatment of hippocampal cells with leptin prevents mitochondrial membrane depolarisation and mitochondrial fragmentation induced by toxic Aβ_1–42_, via a process involving the altered expression of mitochondrial fusion and fission proteins [53]. Interestingly, mitochondrial dysfunction is a common feature in neurodegenerative disorders, and correcting mitochondrial dysfunction is a growing avenue for developing novel therapies for these CNS disorders [54]. Additionally, there is increasing evidence that leptin displays protective actions in other brain disorders. Indeed, exposure to leptin protects against the aberrant brain changes that occur in various animal models of ischaemic stroke. The pathological injury that occurs following ischaemic stroke has been linked to mitochondrial damage and production of oxygen free radicals [55,56]. Leptin treatment not only limits infarct size but also reduces the levels of oxygen free radicals by enhancing the expression of antioxidants and superoxide dismutase [56]. The leptin-driven protective mechanisms have been well-characterised in a variety of models of neuronal cell death. Two key signalling cascades activated downstream of LepRs, namely JAK-STAT3 and PI3-kinase, have been identified as fundamental for leptin-driven neuroprotection [52,57,58,59]. Indeed, in a model of cerebral ischaemic/reperfusion injury, leptin inhibits neuronal apoptosis and other pathological changes via a PI3-kinase-dependent mechanism [60]. Similarly, leptin-driven stimulation of PI3-kinase is vital for leptin’s ability to limit MPP^+^-induced cell death in SH-SY5Y cells [61], and to prevent Aβ_1–42_-induced apoptosis in hippocampal neurons [57]. Furthermore, JAK-STAT3 signalling contributes to leptin’s ability to reduce injury in models of cerebral ischaemic [62,63]. Recent studies have also implicated leptin-driven regulation of microglia in the neuroprotective actions of leptin [64], suggesting that leptin may limit neuronal damage via controlling the pro-inflammatory factors from microglia. However, further studies are required to uncover the precise role of microglia, and the interplay between microglia and the other neuroprotective mechanisms driven by leptin.

## 6. Leptin Has Protective Effects at Synapses in Early AD

Accumulation of amyloid beta (Aβ) and formation of amyloid plaques are well-established pathological features of AD. Treatment with leptin has been shown to decrease expression of β- and γ secretase, two key enzymes that catalyse the cleavage of amyloid precursor protein (APP) into the toxic forms of Aβ [65,66]. Thus, in wild-type H4 cells, treatment with leptin attenuated the expression of several key parts of the γ-secretase enzyme complex, including PS1, PEN2, nicastrin, and APH1B, by up to 50% [66]. Moreover, in the same study it was observed that leptin reduced the levels of Aβ by directly inhibiting the activity of β-secretase, the key enzyme that catalyses production of toxic Aβ_1–42_. Other studies performed in neuronal cells and in AD models that overexpress Aβ [67] found that exposure to leptin decreased the extracellular levels of Aβ by increasing LRP1-dependent uptake of Aβ, thereby reducing the overall amyloid load in the brain. Additionally, leptin reportedly increases the expression of the insulin-degrading enzyme which facilitates the degradation of Aβ [65].

Build-up of neurofibrillary tau tangles is another well-documented pathological feature that occurs in AD. Tau is a microtubule-associated protein that becomes hyper-phosphorylated in AD, due to actions of the serine/threonine kinase, glycogen synthase kinase 3β (GSK3β). Recent reports indicate that the treatment of PC12 neuronal cells with leptin stimulates PI3-kinase which drives the inhibition of GSK3β, ultimately leading to attenuated levels of phosphorylated tau [68,69]. In line with leptin regulating the levels of phosphorylated tau (p-tau), insensitivity or resistance to leptin is linked to enhanced neuronal expression of p-tau in rodents [57]. Moreover, markedly reduced circulating levels of leptin have been detected in rodents with progressive tau-related pathology [70].

In the early stages of AD, exposure to oligomeric Aβ gives rise to impaired functioning of excitatory synapses. In acute hippocampal slices, application of oligomeric Aβ markedly influences hippocampal synaptic plasticity as Aβ blocks the induction of LTP, and it facilitates long-term depression (LTD) [71,72]. Furthermore, in accordance with the pivotal role of AMPA receptor trafficking in synaptic plasticity [33], acute treatment of hippocampal neurons with oligomeric Aβ drives the synaptic removal of AMPA receptors [71]. Studies by Doherty et al. [57] demonstrated that leptin protects against the aberrant synapto-toxic effects of Aβ as the ability of Aβ to block LTP induction is prevented in brain slices treated with leptin. Similarly, treatment with leptin prevented the facilitation of hippocampal LTD induced by Aβ [57]. Moreover, in parallel studies utilising primary hippocampal neurons, the ability of Aβ to remove the AMPA receptor subunit, GluA1 from synapses was blocked after treatment with leptin [57]. In line with the known protective actions of the PI3-kinase signalling cascade, the ability of leptin to prevent the aberrant effects of Aβ on synaptic plasticity and AMPA receptor trafficking involves the stimulation of a PI 3-kinase-dependent pathway [57]. Previous studies have identified that GSK3β inhibitors not only mirror but also occlude the effects of leptin [58,68]. Moreover, as PI 3-kinase activity drives inhibition of GSK3β, it is feasible that GSK3β is a crucial component in leptin’s protective effects against the aberrant effects of Aβ on synaptic function.

Tau is a microtubule associated protein that plays a pivotal role in preserving the stability and flexibility of microtubules, which in turn helps to sustain neuronal structure [73]. Tau is usually highly expressed in axons, but in AD tau becomes hyper-phosphorylated which uncouples tau from microtubules and drives tau from its usual locus in axons into synapses [74]. This in turn ultimately results in impaired excitatory synaptic function and loss of synapses [75]. It is known that the detrimental effects of tau on hippocampal excitatory synapses correlate well with the early cognitive deficits reported in AD [76,77]. Consequently, identifying ways to prevent the synapto-toxic effects of tau in the early pre-clinical stages of AD is likely to have beneficial effects in AD. Indeed, our recent immunocytochemical studies support this, as the treatment of a cellular model of tau-related synaptic dysfunction with leptin prevented tau mis-location to dendrites and synapses [58]. In this model, chronic treatment with Aβ oligomers drives the activation of GSK-3β, which in turn promotes the phosphorylation of tau [78] and the subsequent delivery of tau to synapses [74,75]. There is good evidence that GSK-3β-dependent phosphorylation of tau at serine 396 (Ser396) is significantly elevated in AD and that phosphorylation at the tau Ser396 site is crucial for the aberrant movement of tau to synapses [58]. Our recent studies demonstrated that leptin prevented tau phosphorylation at Ser396, thereby inhibiting the synaptic insertion of tau [58]. In line with previous work that uncovered that leptin-driven inhibition of GSK-3β reduces tau phosphorylation in neuronal cells [68], the capacity of leptin to restrict tau movement to hippocampal synapses also involves PI 3-kinase-driven inhibition of GSK-3β [58].

Previous studies have identified that tau phosphorylation and its resulting delivery to synapses leads to impaired synaptic function due to the synaptic removal of AMPA receptors [74]. In accordance with this, trafficking of tau to synapses is correlated with attenuated synaptic expression of GluA1-containing AMPA receptors, thereby confirming that tau phosphorylation prompts AMPA receptor endocytosis and removal from synapses [58]. Consistent with this, the direct application of oligomeric tau to hippocampal neurons also reduced the surface expression of GluA1, supporting the notion that oligomeric tau drives the internalisation of AMPA receptors [58]. Our recent studies have shown that leptin protects against these tau-dependent synaptic impairments, as AMPA receptor removal from synapses induced by either tau phosphorylation or the direct administration of oligomeric tau is prevented by prior treatment with leptin [58]. Moreover, in acute brain slices, exposure to leptin blocked the oligomeric tau-driven inhibition of activity-dependent LTP at hippocampal SC-CA1 synapses [58]. Thus, leptin not only attenuates GSK3β-driven phosphorylation of tau, which in turn limits tau trafficking to synapses, but leptin also restricts the tau-driven synaptic abnormalities by preventing synaptic removal of AMPA receptors and inhibiting hippocampal synaptic plasticity. Increasing evidence supports a pivotal role for abnormal build-up of tau in the cognitive decline associated with AD [76]. Consequently, leptin’s capacity to restrict the aberrant synapto-toxic effects of tau has important implications for leptin’s protective role in neurodegenerative disorders like AD.

## 7. Leptin Has Pro-Cognitive Effects in AD Models

There is now good evidence from studies using obese rodents (*db*/*db* mice; *fa*/*fa* rats) that leptin plays a crucial role in learning and memory processes, as impaired spatial memory is observed in rodents with LepR mutations which result in leptin insensitivity [14]. In addition to enhancing memory in wild-type rodents, significant evidence now indicates that exposure to leptin has pro-cognitive actions in rodent models of AD. Thus, improvements in novel object recognition tasks, and the cue fear conditioning test were detected in CRND8 transgenic mice (TgCRND8) following treatment with leptin for 8 weeks [47]. Intra-cerebroventricular administration of a leptin viral gene into APP/PS1 transgenic mice also rescued Aβ-associated memory deficits [67], whereas spatial memory impairments induced in rats by ICV administration of Aβ were alleviated following chronic leptin treatment [79]. On the flip side, deficiencies and/or loss of leptin are reported to exacerbate cognitive decline in AD, as cognitive function is reportedly much poorer in double-mutant (APP(+)-*ob*/*ob*) mice compared to APP(+) mice [80].

## 8. Therapeutic Potential of Leptin and Leptin-Derived Peptides

Clinical evidence has identified that treating leptin-deficient patients with leptin results in improved cognitive function [81,82]. Although it is well-documented that leptin-based therapies can be safely used in humans, there has been a paucity of clinical studies evaluating the effectiveness of leptin in AD patients. However, what is clear is that it is unlikely that all AD patients would benefit from leptin-based therapies. Indeed, individuals with leptin-resistance due to mid-life obesity are unlikely to see any benefit, whereas those with low circulating leptin levels may be highly responsive to leptin replacement. Indeed, metreleptin, which is a modified synthetic form of human leptin, has already been approved for treatment of lipodystrophy [82]. Moreover, clinical studies have identified beneficial effects of metreleptin on cognitive function in individuals with low leptin levels due to lipodystrophy [83,84]. Marked improvements in cognition have also been observed following metreleptin treatment in anorexia patients, who also display low circulating levels of leptin [85,86]. Consequently, as there is good evidence that leptin-based therapies have therapeutic efficacy in individuals with low leptin levels, it feasible that the use of leptin-based therapies will have beneficial effects in AD, but the usefulness may be limited to a specific cohort of AD patients.

As leptin has wide-spread neuronal actions, it is key that steps are taken to circumvent or reduce the likelihood of potential unwanted side effects. Accordingly, developing leptin-based drugs that are modified to target the specific brain regions, such as the hippocampus and cortex, that degenerate in AD is one potential way forward. Additionally, fragments of the whole leptin molecule (eg Leptin_116–130_) are not only bioactive [87] but also recapitulate the hippocampal actions of leptin [88], indicating that small molecule leptin mimetics could have benefits in AD. Indeed, our recent work has identified that leptin_116–130_ prevents the aberrant effects of Aβ on hippocampal synaptic plasticity, AMPA receptor trafficking, and neuronal toxicity [88]. Moreover, peripheral administration of leptin_116–130_ mirrored the pro-cognitive actions of whole leptin as mice treated with leptin_116–130_ displayed improved performance in behavioural tests of episodic-like memory [88]. Our more recent studies have observed similar results using smaller leptin-based hexamers, such that four out of the eight hexamers derived from leptin_116–130_ were found to replicate the full spectrum of pro-cognitive and neuroprotective actions of leptin [89]. Interestingly, a small molecule leptin mimetic, MA-[D-Leu-4]-OB3 has also been reported to enhance episodic memory in rodent models of diabetes [90]. Collectively, this confirms not only that leptin_116–130_ and the bioactive hexamers can readily access the brain and influence hippocampal function, but these leptin-based molecules also offer potential novel avenues for developing new drugs to treat AD in the future. Although the identification of leptin-based molecules as potential therapeutic targets is a promising advance, there are still some challenges to be overcome before these agents could be used in AD patients. Significant pre-clinical studies are still needed, before leptin-derived agents could be used in clinical trials. Indeed, it is vital that in vivo studies are undertaken to determine the potential efficacy of the various bioactive leptin fragments/hexamers in rodent models of AD. Further studies are required to evaluate the pharmacokinetic properties of the leptin-based molecules, as it is not clear to what extent these agents are metabolised after peripheral or oral administration, and what proportion of the parent molecule reaches the brain target sites.

## 9. Conclusions

**Figure 1:**
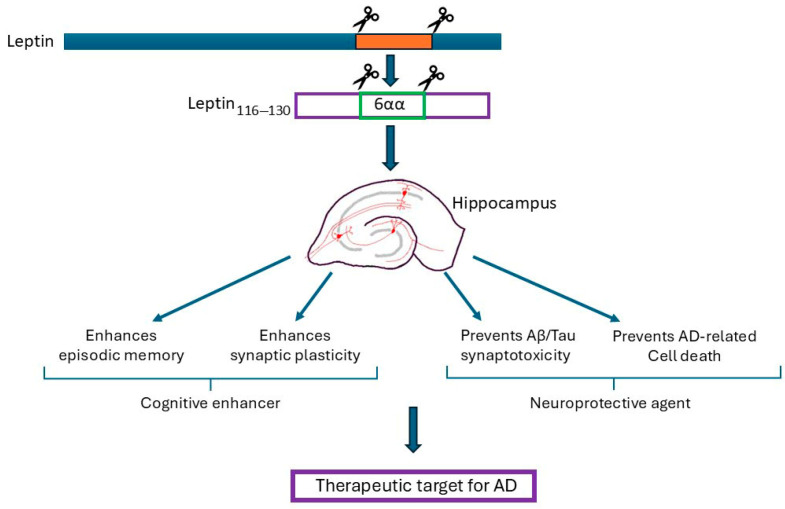
The summary of the potential therapeutic benefits of leptin and leptin-derived fragments. The hippocampus is a key brain target for the actions of leptin. Exogenous application of leptin results in pro-cognitive effects as leptin facilitates synaptic plasticity and AMPA receptor trafficking; the key cellular changes required for hippocampal-dependent learning and memory. The administration of leptin into rodents also results in enhanced performance in various types of memory, including episodic-like memory which is the first memory that is lost in AD. In various models of AD, treatment with leptin prevents the unwanted toxic synaptic effects of β-amyloid (Aβ) and p-tau, thereby protecting against the acute synaptotoxic effects that occur in early AD. Moreover, leptin limits the chronic neurotoxic effects of Aβ and tau, with enhanced neuronal viability observed after treatment with leptin. Collectively, studies using cellular and rodent models of AD have identified that leptin-based treatments have pro-cognitive and neuroprotective actions, and therefore have potential therapeutic benefit in AD. Moreover, the potential cognitive enhancing and neuroprotective actions of whole leptin are replicated by smaller leptin fragments (leptin_116–130_), and leptin hexamers (eg leptin_116–121_).

The hippocampus is now established as a key target site for the hormone leptin in the CNS, with significant evidence indicating that leptin has pro-cognitive properties. It is well-documented that leptin can modify excitatory synaptic strength at SC-CA1 and TA-CA1 synapses, which has important implications for its role in hippocampal learning and memory. Moreover, as the hippocampus is a key site for degeneration in AD, the regulatory actions of leptin are also crucial in age-related neurodegenerative disorders associated with cognitive deficits. Indeed, clinical evidence increasingly supports a link between brain metabolic health and AD, with an identified association between the circulating levels of leptin and the risk of developing AD later in life. Furthermore, significant evidence from studies using cellular and rodent models of AD indicates that leptin-based treatments have pro-cognitive and neuroprotective actions, thereby signifying that targeting the leptin system may be advantageous in the treatment of AD (see Figure 1). However, the use of leptin therapeutically is liable to be limited by metabolic status, as leptin is unlikely to be effective in individuals with resistance to leptin. Consequently, further clinical evaluation of the potential feasibility and efficacy of using leptin-based treatments in AD is needed.

## Data Availability

No new data were created or analyzed in this study. Data sharing is not applicable to this article.
